# Uterine Rupture in Pregnancy following Fall from a Motorcycle: A Horrid Accident in Pregnancy—A Case Report and Review of the Literature

**DOI:** 10.1155/2015/715180

**Published:** 2015-10-21

**Authors:** Wondimagegnehu Sisay Woldeyes, Demisew Amenu, Hailemariam Segni

**Affiliations:** Department of Obstetrics and Gynecology, School of Medicine, College of Health Sciences, Jimma University, P.O. Box 378, Jimma, Ethiopia

## Abstract

Uterine rupture is one of the most catastrophic complications during pregnancy. It is a rare complication in developed countries but a frequent cause of maternal and perinatal morbidity and mortality in Africa. Uterine rupture occurs in 1.6% of patients suffering blunt abdominal trauma. Here we report a unique case of complete fundal rupture of the unscarred uterus following fall from motorcycle in 39-week-pregnant mother who was managed with total abdominal hysterectomy and left salpingo-oophorectomy and survived, though fetus died before intervention. We also reviewed similar cases reported from different parts of Africa. This is a preventable complication had the woman been properly instructed on transportation safety during her antenatal care visits.

## 1. Introduction

Uterine rupture is a serious obstetric complication, with high morbidity and mortality, particularly in less and least developed countries. With ready access to obstetric care, including caesarean section for obstructed labor, rupture of the unscarred uterus should be rare [1]. Nevertheless, it is still a major public health problem in developing countries in general and in Ethiopia uterine rupture and obstructed labor are responsible for 36% of maternal deaths in the last decade [2]. Rupture of the uterus can follow oxytocin stimulation, uterine scar, obstructed labor, and rarely blunt abdominal trauma [3].

Motor vehicle accidents, domestic violence, and falls are the most common causes of blunt trauma during pregnancy [4, 5]. Motor vehicle accidents involving pregnancy were associated with elevated rates of adverse pregnancy outcomes, including preterm birth, stillbirth, placental abruption, and rupture of fetal membrane. In all pregnant mothers hospitalized for trauma to a teaching hospital in Nigeria, uterine rupture occurred in 1.6% [4]. Although there are few case reports of uterine rupture following a fall from motorcycle, there are none from Ethiopia [6–9]. Here we report a rare case of uterine rupture following a fall from a motorcycle at 39 weeks of pregnancy, where the mother's life was saved, though fetus died before intervention.

## 2. Case Report 

A 30-year-old patient, gravida 6 para 5 (all alive), at 39 weeks of gestation was admitted to our hospital 4 hours after she sustained motorcycle accident. She was a passenger on motorcycle sitting behind the driver. The driver suddenly changed direction as pedestrian got in their way; she lost balance and fell flat on the ground. Following the accident, she started to experience severe abdominal pain and faints on attempt to get up. There was no vaginal bleeding or passage of liquor. She had no other apparent injuries and was conscious without vomiting. She had antenatal care at the local health center. All previous deliveries were vaginally at home. She had no gynaecological surgery.

On physical examination, she was confused with blood pressure of 80/40 mmHg, pulse rate of 132 beats per minute; she was pale and had cold extremities. She had a bruise of 4 by 5 centimeters over her abdomen just above umbilicus and the abdomen was tender all over. There were signs of intra-abdominal fluid collection with easily palpable fetal parts. Per vaginal examination the cervix was closed; she had no vaginal bleeding. Sonographic examination revealed peritoneal fluid collection and empty enlarged uterus and the fetus in abdominal cavity had no heart activity.

Uterine rupture was diagnosed and resuscitation started with crystalloid. Blood was sent for cross match and laparotomy was done with the consultation of senior obstetrician. At operation, about 2500 mL blood was present in the peritoneal cavity and the fetus had apparently recently died. The placenta was in the peritoneal cavity and other abdominal organs were normal. Blood, fetus, placenta, and membranes were removed from peritoneal cavity.

**Figure 1:**
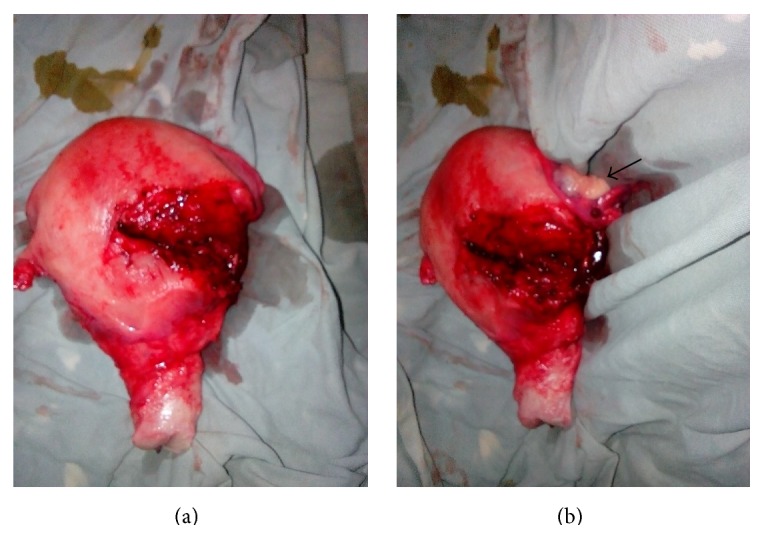
The rupture on total abdominal hysterectomy specimen anterior (a) with attached left ovary left lateral (b) views. The uterine rupture was located on the left upper segment (a) extending laterally causing detachment of left ovary from pelvic side wall (arrow) (b).

There was a complete transverse tear measuring 10 centimeters in the left upper segment of the uterus, extending laterally to involve the left uterine vessels. At the same side the round and infundibulopelvic ligaments were torn. Total abdominal hysterectomy and left salpingo-oophorectomy were done (Figure 1). She received transfusion with 3 units of whole blood and her postoperative period was uneventful. She was discharged on her seventh postoperative day in good condition.

## 3. Discussion 

Safety is critical in any transport system; this is specially an area of concern when it comes to transporting pregnant women. Trauma affects 6 to 7 percent of pregnancies in the United States and is the leading cause of nonobstetric maternal death [10]. In developed countries motor vehicle accidents are the leading cause and account for up to 80% of trauma in pregnancy, followed by falls and assaults [5]. In reports from developing countries on the other hand, physical assault was the cause of injury observed in 46% trauma in pregnancy, whereas road traffic accidents and falls accounted for injuries in 30.2% and 14.3%, respectively [4]. Motorcycles were involved in as low as 0.9% in a population based study [5] to as high as 83.3% of motor vehicle accidents during pregnancy in hospital based study [4] indicating high likelihood of severe injury and hospitalization after motorcycle accidents.

Although motor vehicle crashes are responsible for most severe maternal injuries and fetal losses from trauma, pregnant women are less aware of measures to decreasing maternal and fetal injury and mortality after motor vehicle accidents [10]. In this case, although the patient survived a horrible accident, she lost her baby while traveling on a motorcycle. This could have been prevented by proper instruction on transportation options and safety during her antenatal care visits.

The fetal and neonatal outcomes in the pregnancies that are delivered during motor vehicle accident admissions were poor. About a third ended in a perinatal death [5]. With uterine rupture perinatal death can approach 100% unless immediate action toward delivery is taken [11]. In our patient fetal death was inevitable, as she presented late in shock with fetus and placenta expelled into abdominal cavity.

There is no universal standard for management of uterine rupture. Total or subtotal abdominal hysterectomy, repair, or repair with tubal ligation can be considered depending on the location, hemodynamic status of the patient, future fertility desire [12], availability of blood products, and experienced surgeon. Repair of the uterus is possible in the majority of women. In others, haemorrhage from an extension of the rupture into the broad ligament or extensive damage to the uterus requires hysterectomy [12].

There are case reports on uterine rupture in pregnancy following motor vehicle accidents involving motorcycle from other parts of Africa and are managed differently [6–9]. We reviewed four cases reported from Rwanda, Morocco, and Nigeria (Table 1). Three of the cases were less than 28 weeks pregnant and two were multigravid. The uterine rupture was on the fundus in two of the cases and left lateral and posterior for the other two. Three of the cases were hemodynamically unstable at presentation. Total abdominal hysterectomy was done for a case of posterior rupture and the other three were managed with repair of the rupture. Only one of the fetuses survived and there was also one maternal death two days after rupture repair. In our patient considering the fact that she is multigravid and hemodynamically unstable, with fundal rupture extending to left adnexae, we managed with total abdominal hysterectomy and left salpingo-oophorectomy.

In conclusion, this is a preventable complication had the woman been properly instructed on transportation safety during her antenatal care visits. Pregnant women should be informed about safety of different options of transportation and instructed on precautions to avoid injury during motor vehicle accidents as part of birth preparedness and complication readiness advice during antenatal care. As mentioned above over 83% of hospitalization following motor vehicle accidents during pregnancy involved a motorcycle [4] and serious consequences to the fetus and mother occurred in all trimesters of pregnancy (Table 1). Therefore, its use for transporting pregnant mothers should generally be discouraged.

## Figures and Tables

**Table 1:** Cases of uterine rupture following motorcycle accident.

Case	Age	G-P	GA	Site	Shock	Management	Fetus	Reference
1	25	G1	19	Fundal	No	Repair	Died	[6]
2	33	G6P5	26	Posterior	Yes	TAH	Died	[7]
3	26	G6P5	38	Left lateral	Yes	Repair	Saved	[8]
4	36	UK	12	Fundal	Yes	Repair (MD)	Died	[9]

G-P: gravida-para: UK: unknown; GA: gestational age; MD: maternal death.
